# Data quality predicts care quality: findings from a national clinical audit

**DOI:** 10.1186/s13075-020-02179-y

**Published:** 2020-04-17

**Authors:** Mark Yates, Katie Bechman, Elaine M. Dennison, Alexander J. MacGregor, Jo Ledingham, Sam Norton, James B. Galloway

**Affiliations:** 1 The Centre for Rheumatic Diseases, School of Immunology, Infection & Inflammatory Disease, King’s College London, Room 3.46 Weston Education Centre, Cutcombe Road, London, SE5 9RJ UK; 2 MRC Epidemiology Unit, University of Southampton, Southampton, UK; 3 Norwich Medical School, University of East Anglia, Norwich, UK; 4 Department of Rheumatology, Queen Alexandra Hospital, Portsmouth, UK; 5 Institute of Psychiatry, Kings College London, London, UK

**Keywords:** Rheumatoid arthritis, Missing data, National clinical audit, Care quality, Methodology

## Abstract

**Background:**

Missing clinical outcome data are a common occurrence in longitudinal studies. Data quality in clinical audit is a particular cause for concern. The relationship between departmental levels of missing clinical outcome data and care quality is not known. We hypothesise that completeness of key outcome data in a national audit predicts departmental performance.

**Methods:**

The National Clinical Audit for Rheumatoid and Early Inflammatory Arthritis (NCAREIA) collected data on care of patients with suspected rheumatoid arthritis (RA) from early 2014 to late 2015. This observational cohort study collected data on patient demographics, departmental variables, service quality measures including time to treatment, and the key RA clinical outcome measure, disease activity at baseline, and 3 months follow-up. A mixed effects model was conducted to identify departments with high/low proportions of missing baseline disease activity data with the results plotted on a caterpillar graph. A mixed effects model was conducted to assess if missing baseline disease activity predicted prompt treatment.

**Results:**

Six thousand two hundred five patients with complete treatment time data and a diagnosis of RA were recruited from 136 departments. 34.3% had missing disease activity at baseline. Mixed effects modelling identified 13 departments with high levels of missing disease activity, with a cluster observed in the Northwest of England. Missing baseline disease activity was associated with not commencing treatment promptly in an adjusted mix effects model, odds ratio 0.50 (95% CI 0.41 to 0.61, *p* < 0.0001).

**Conclusions:**

We have shown that poor engagement in a national audit program correlates with the quality of care provided. Our findings support the use of data completeness as an additional service quality indicator.

## Background

National clinical audits (NCAs) are a key lever to improve quality of care and limit unwarranted variation. The National Lung Cancer Audit and the Sentinel Stroke National Audit Programme (SSNAP) are examples of how care can be improved through national audit [1, 2]. In order to assess care quality, NCAs rely on metrics that can be split into two distinct groups:

1. *Process measures*. Typically a guideline recommendation that applies a threshold of quality to a particular aspect of care delivery [3], for example, time to surgery in the hip fracture database [4] or time to thrombolysis in SSNAP [1];
2. *Clinical outcome measures*. Quantifiable changes in health status as a result of an intervention [5], for example, blood glucose levels in the National Diabetes Audit [6] or disease activity scores in the National Early inflammatory arthritis audit [7].

Missing or incomplete data are a common finding in any longitudinal dataset, particularly in NCAs that, by and large, rely on clinician time and goodwill for data collection. Process measures tend to have higher data completeness as they are simple to collect from electronic health records. Clinical outcomes measures are more challenging to collect, often requiring blood tests, specific examination findings, or calculation of scores derived from multiple metrics, and so are more likely to have lower data completeness.

Rheumatoid arthritis (RA) is primarily managed in the outpatient setting with multiple follow-up appointments. This increases the likelihood of missing outcome data. Despite this, patterns of missing data are frequently not reported in RA observational studies [8].

It is not known if departments with higher levels of incomplete clinical outcome data are delivering lower quality care. We hypothesised that the completeness of key outcome data in a national clinical audit could predict departmental performance.

The objectives of this study are to utilise an NCA dataset to:

1. Describe departmental level variations in outcome data quality across the National Health Service (NHS) in England and Wales;
2. Investigate if completeness of a clinical outcome measure correlates with process measures.

## Methods

### Sample

In 2014, the Healthcare Quality Improvement Partnership (HQIP)-commissioned National Clinical Audit for Rheumatoid and Early Inflammatory Arthritis (NCAREIA) was launched. This NCA sought to assess the quality of early inflammatory arthritis (EIA) care delivered and clinical outcomes. All rheumatology departments across England and Wales were mandated to participate. Data were collected at departmental level from February 2014 to October 2015 via an online portal. Data were only reported from departments that contributed > 5 participants to each analysis, in line with the audit’s low number reporting policy to maintain anonymity.

Patients aged > 16 years referred to a rheumatology department with symptoms consistent with EIA were eligible for inclusion, but the analyses in this study only required data for patients with symptoms consistent with RA. This was defined as any patient with a new onset of inflammatory arthritis, longer than 6 weeks duration, and either a positive rheumatoid factor (RF), anti-cyclic citrullinated protein (anti-CCP) antibody, or a swollen joint count of five or more. Data were collected at baseline appointment and at a 3-month follow-up.

### Data

Patient demographic variables collected at baseline included age, gender, smoking status, ethnicity, work status, and the first three characters of postcode.

A proxy rank of the index of multiple deprivation (IMD) was calculated from partial patient postcodes by identifying all lower super output areas (LSOAs) covered by each postcode. The corresponding IMD scores were then averaged for each patient, allowing calculation of an estimation of deprivation across the cohort.

Departmental variables collected included staffing levels, catchment population, and dedicated EIA clinic presence. The process measures collected included (1) number of days from primary care review to referral to rheumatology care (referral time), (2) number of days from referral to rheumatology review (review time), and (3) number of days from referral to treatment commencement (treatment time).

Referral and review times were calculated at baseline appointment for all patients. Data from the 3 months follow-up was used to calculate treatment time for those who were not commenced on treatment at their baseline appointment. The process measures were collected in accordance with national guidelines for suspected inflammatory arthritis care [9].

Clinical outcome measures collected at baseline and 3 months follow-up included tender and swollen joint counts, patient-reported visual analogue scale of symptom severity, blood erythrocyte sedimentation rate (ESR), and C-reactive protein (CRP) levels.

The clinical outcome measures were utilised to calculate disease activity scores (DAS28), a multi-component score of disease activity in RA [10]. DAS28 is a key clinical outcome measure, utilised widely across rheumatology services to assess response to therapy. The score is calculated from tender and swollen joint counts, a patient assessment of overall symptom severity on 100-mm visual analogue scales, and blood inflammatory marker levels (ESR or CRP), with higher scores indicating greater disease severity. If patients had both an ESR and CRP level, the ESR level was preferentially used to calculate their DAS28.

### Statistical analysis

#### Characterising departmental variation in data completeness

A mixed effects model was conducted to identify departments with high or low proportions of missing baseline DAS28 data, adjusting for case mix. Departments who had recruited more than five patients were included in the model.

Missing baseline DAS28 data was the outcome variable with patient-level variables included as fixed effects (age, gender, work status, area level social deprivation, smoking status; ethnicity, symptom duration, antibody status) and department included as a random effect. The random effect captures the difference between the departmental proportion of incomplete data and the overall proportion of incomplete data in the sample. The estimated proportions from the model are empirical Bayes estimates, which, following the logic of Bayesian inference, is a ‘shrinkage’ estimator exploiting additional information that typically moves the estimate towards the overall proportion for the sample. Shrinkage estimates have several desirable properties, including greater precision and accounting for larger random variation observed at smaller centres [11].

The observed and model-estimated departmental proportions of missing baseline DAS28 data were plotted on a caterpillar graph, with 95% confidence intervals (CI) for the predicted values, to allow a ranking of departments by proportions of missing data.

A department was considered to have a high proportion of missing data if their predicted 95% CI lower bound was greater than the overall mean. Similarly, a department was considered to have a low proportion of missing data if their predicted 95% CI upper bound was less than the sample mean. The locations of the departments with high and low levels of missing data were mapped using the grmap software package [12] in Stata 15 to give a national representation.

#### Missing clinical outcome data as a predictor of service quality

To test the hypothesis that completeness of key outcome data is a usable surrogate for departmental performance, mixed effects modelling was performed with the department providing care included as a random effect. Incomplete baseline DAS28 was the independent variable, with treatment time as the dependent variable.

Treatment time was used in preference to referral time, as the latter is an assessment of primary care rather than rheumatology department performance. Shorter treatment times are considered a key marker of service quality in early RA care [13]. Analyses from the latest EIA NCA annual report show that shorter treatment times correspond to better clinical outcomes [14].

Treatment time was converted to a binary variable indicating whether treatment commenced within 90 days of referral to rheumatology specialist care. The model was first performed unadjusted for covariates with reporting of odds ratios, *p* values, and 95% CI for the primary hypothesis test.

The model was repeated adjusting for patient level covariates selected a priori: age, gender, work status, area level social deprivation, smoking status, ethnicity, symptom duration, rheumatoid, and anti-CCP antibody status. The adjusted model was then repeated with further adjustment for departmental characteristics selected a priori: specialist nurse and consultant staffing levels per head of catchment population; presence or absence of a dedicated EIA clinic, and departmental proportions of missing DAS28 data. Model fit was assessed by comparing area under the curve values (AUC) for observed and predicted performance data.

As analyses were exploratory in nature, no correction for multiple hypothesis testing was performed. All analyses were conducted using Stata 15 statistical software package.

## Results

A total of 6205 patients with a diagnosis of RA and complete treatment time data were recruited from 136 departments across England and Wales. Table 1 details baseline and demographic characteristics, process measures, clinical outcome measures, and proportions of missing data. There were substantial proportions of incomplete DAS28 data at baseline with 2130/6205 (34.3%) missing data.

**Table 1:** Baseline characteristics and quality of care received, including levels of missing data

***N*** = 6205		Number missing (%)
**Age, mean (SD)**	58.2 (15.2)	37 (0.5)
**Female, %**	63.7	37 (0.5)
**IMD rank, median (Q1, Q3)**	639 (300 to 1021)	683 (11.0)
**White European, %**	90.8	1423 (23.0)
**Current smoker, %**	22.3	1170 (18.6)
**Full time paid employment, %**	39.3	1236 (20.0)
**Seropositive, %**	77.8	1594 (25.7)
**Baseline DAS28, mean (SD)**	5.1 (1.4)	2130 (34.3)
**Follow-up DAS28, mean (SD)**	3.5 (1.5)	3864 (62.3)
**Change in DAS28, mean (SD)**	1.7 (1.6)	4222 (68.0)
**Rheumatology Departments**	136	0
**Symptom duration in days, median (Q1, Q3)**	103 (53 to 223)	59 (1.0)
**Referral within 3 days, %**	17.0	0
**Review within 21 days, %**	37.2	0
**Treatment within 90 days, %**	60.1	0

*SD* standard deviation, *IMD* index of multiple deprivation, *DAS28* disease activity score 28

Patients who were positive for rheumatoid factor and/or anti-citrullinated c-peptide were considered seropositive

Table 2 provides further detail on the missing components of baseline DAS28.

**Table 2:** DAS28 missing data table with components breakdown

***N*** = 6205	Number missing at baseline (%)
**Tender joint count**	370 (6.0%)
**Swollen joint count**	372 (6.0%)
**Patient global VAS**	1012 (16.3%)
**ESR**	2075 (33.4%)
**CRP**	1625 (26.2%)
**ESR and CRP**	472 (7.6%)

*VAS* visual analogue scale, *ESR* erythrocyte sedimentation rate, *CRP* C-reactive protein

There were demographic differences between patients with complete and incomplete baseline DAS28 data (see Table 3). Those with incomplete baseline DAS28 data were younger, less likely to smoke, more likely to be in paid work, less likely to be RF or anti-CCP antibody positive, and had longer symptom duration prior to presentation. Amongst those with incomplete baseline DAS28, 50.3% were commenced on treatment within 90 days, compared to 65.3% in those with complete DAS28 data.

**Table 3:** Baseline characteristics, disease activity, and quality of care received stratified by complete and incomplete baseline DAS28 data

	Complete baseline DAS28 data	Incomplete baseline DAS28 data	***p*** values
	(*N* = 4075)	(*N* = 2130)	
**Age, mean (SD)**	59.0 (14.8)	56.6 (15.8)	< 0.0001
**Female**	64.1%	62.8%	0.3
**IMD rank, median (Q1, Q3)**	638 (306, 1021)	639 (270, 1021)	0.3
**White European**	90.6%	91.3%	0.5
**Current Smoker**	23.5%	19.8%	0.003
**Full time paid employment**	36.8%	40.7%	0.008
**Seropositive**	83.0%	65.3%	< 0.0001
**DAS28 at follow-up, mean (SD)**	3.5 (1.5)	3.6 (1.5)	0.3
**Symptom duration in days, median (Q1, Q3)**	98 (52, 208)	114 (56, 256)	0.0001
**Referral letter states EIA**	91.2%	88.8%	0.002
**Referral within 3 days**	16.5%	18.0%	0.1
**Review within 21 days**	36.9%	36.7%	0.6
**Treatment within 90 days**	65.3%	50.3%	< 0.0001

*SD* standard deviation, *IMD* index of multiple deprivation, *DAS28* disease activity score 28, *EIA* early inflammatory arthritis

Patients who were positive for RF and/or anti-CCP were considered seropositive

### Characterising departmental variations in data completeness

**Fig. 1:**
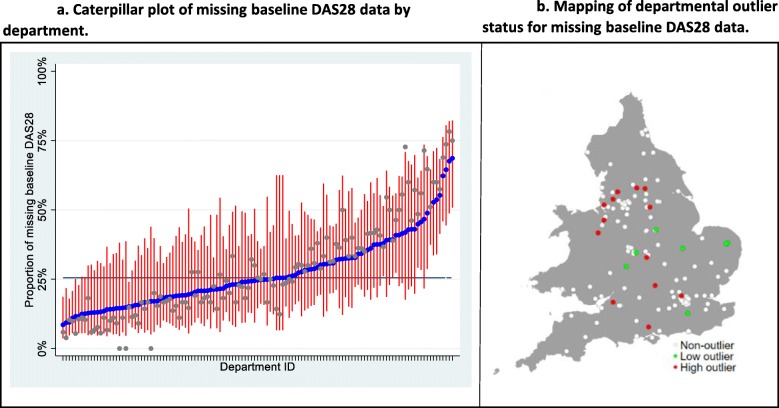
Caterpillar plot and map characterising departmental variations in data completeness. **a** The grey markers are the observed departmental proportions of missing baseline DAS28 data. The blue markers are the predicted departmental proportions after adjustment for case mix and applying a shrinkage to account for the small overall sample size, with 95% confidence intervals in red. The black horizontal line represents the overall sample mean. The further to the right along the *x*-axis indicates worse performance by a department, i.e. a greater proportion of missing DAS28 data. **b** Locations of outlier departments mapped across England and Wales. Red markers represent departments with high outlier levels of missing baseline DAS-28. Green markers represent low outliers

Mixed effects modelling to identify departments with outlier levels of missing DAS28 data was conducted. The model identified 13 departments with high levels of missing data and 7 with low levels. The case-mix adjusted departmental effects are plotted in Fig. 1a. The analysis demonstrates wide variation across departments.

The model has accounted for sample size and case mix, shrinking estimates towards the grand mean as a result of them being empirical Bayes estimates, with the level of shrinkage dependent on the number of observations for each department.

The locations of outlier departments were mapped, displayed in Fig. 1b, with red markers indicating departments with high outlier levels of missing baseline DAS28 data. Geographic clustering of high outlier departments is visible in the North West region.

### Missing clinical outcome data as a predictor of service quality

**Fig. 2:**
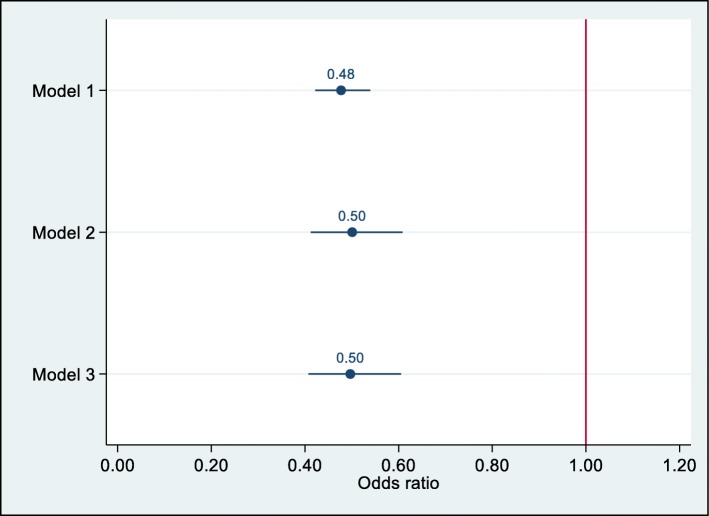
Mixed effects modelling of missing baseline DAS-28 data as a predictor of prompt treatment commencement. Model 1 (*N* = 6205) is unadjusted. Model 2 (*N* = 2979) is adjusted for patient covariates: age, gender, work status, socioeconomic position, smoking status, ethnicity, symptom duration, rheumatoid, and anti-CCP antibody status. Model 3 (*N* = 2943) is adjusted for patient covariates and the following departmental characteristics: specialist nurse and consultant staffing levels per head of catchment population, presence or absence of a dedicated EIA clinic, and departmental proportions of missing DAS28 data. Department was included as a random intercept in all three models

Logistic regression models were constructed to assess if missing baseline DAS28 data predicted timely treatment commencement (within 90 days of referral). In mixed effects models, missing baseline DAS28 was associated with not commencing treatment promptly, with odds ratios of 0.48 (95% CI 0.42 to 0.54, *p* < 0.0001) in the unadjusted model and 0.50 (95% CI 0.41 to 0.61, *p* < 0.0001) in the model adjusted for patient level factors. The association was maintained after adjustment for department level factors, odds ratio 0.50 (95% CI 0.41 to 0.61, *p* < 0.0001). A comparison of the models is presented in Fig. 2.

Staffing levels had a substantial impact on the estimates with higher nursing levels and, paradoxically, lower consultant levels associating with a greater chance for prompt treatment initiation.

Sensitivity analyses were conducted to investigate the impact of staffing levels on data completeness. Mixed effects modelling with nurse and consultant densities as a predictor of missing baseline DAS28 gave odds ratios of 1.11 (95% CI 0.91 to 1.34) and 0.97 (95% CI 0.76 to 1.25) respectively, suggesting staffing levels were not independent predictors of missing DAS28.

## Discussion

These data from an outpatient-based NCA identify a strong relationship between clinical outcome data completeness and care quality, maintained after extensive adjustment for patient and department level factors. The relationship suggests missing baseline disease activity data are an indirect indicator for service quality in RA care.

By using a mixed effects model in our analyses, we have been able to account for case mix and volume of activity in our estimates of data quality. As a result, we have robustly identified departments that substantially deviate from expected data return levels, accounting for confounding due to case-mix and potential small sample effects [11].

There was broad variation in departmental completeness of baseline disease activity data. High levels of incomplete clinical outcome measures detrimentally affect the face validity of NCA findings. Process measures, such as treatment time in NCAREIA, tend to have lower levels of missing data, good precision, and a lower risk of bias. Despite this, clinicians may place greater emphasis on clinical outcome measures even when the data are incomplete [15]. This is perhaps explained by clinician familiarity with the clinical outcome measures from trials within the field, in contrast to the paucity of studies that evaluate process measures in healthcare.

There appeared to be a cluster of departments with high degrees of missing data in the North West region. The purpose of mapping performance was to seek evidence for regional clustering. The mapping presented suggests that clustering may be present, and this warrants further investigation. Clinical departments often share networks within their locality, with common training programs and regional education events. If regional trends in performance are apparent, and genuine, these provide an opportunity for a collective service improvement program.

There were striking differences between patients with complete and incomplete DAS28 data, suggesting the data are missing not at random. The drivers behind missing DAS28 data warrant further discussion. Participation in NCAREIA was mandatory, but still relied on clinician goodwill for data entry. It is likely that clinicians put greater emphasis on collecting data for patients with ‘typical’ or severe symptoms of RA. This is supported by the higher proportion of seropositive patients in those with complete DAS28 data.

Missing data are a challenge in any observational dataset, particularly in NCAs that rely on clinician goodwill for case ascertainment and data collection. HQIP, responsible for the commissioning of NCA activity within the NHS in England, recommend that missing data can be reduced, by rationalising of data collection, so only the most important information is sought; mitigated, via linkage to alternative data sources to ‘backfill’ data gaps; and imputed, utilising statistical modelling [16].

Rationalisation of data collection is a key component in the design of an NCA. A balance must be struck between the desire to collect as much information as possible and the challenge for clinicians tasked with recruiting patients while running busy services. Data linkage to fill in data gaps, for example using the clinical practice research datalink of primary care data to attain patient demographic details, is in theory possible but in practice not feasible as this will require additional approvals to access and link alternative data sources. Therefore, data imputation is often required to allow analysis without omitting individuals with incomplete records, which potentially introduces selection bias. A widely used principled approach to missing data is multiple imputation, which estimates a range plausible missing values [17]. It is now widely used in the published literature and has been utilised in NCAREIA analyses [18].

The impact of missing clinical outcomes in NCAs is often only partially addressed [19, 20], increasing the likelihood of biased findings. All NCAs should have a robust missing data plan in keeping with HQIP recommendations. The findings presented here suggest that in circumstances where missing clinical outcome data cannot be avoided, it may have utility as a surrogate for service performance.

Work on other NCAs has highlighted non-participation as a predictor for departments that deliver lower quality care [21], but this study is the first to highlight the utility of partial participation, submitting incomplete data, as a predictor capable of identifying departments with outlier performance.

The strengths of this study are that it was conducted using a large dataset with missing data characteristics similar to other NCAs. Analyses were extensively adjusted for patient and departmental covariates, with department included as a random effect in the model to account for the hierarchy of the dataset. The findings identified that (1) the degree of missing clinical outcome data varies broadly across departments, allowing the detection of outliers, and (2) missing clinical outcome data associates with service quality. The combination of these findings supports the utility of missing outcome data as a usable proxy for service quality.

These findings do not in themselves support the utilisation of missing outcome data across NCAs. Further work by the respective methodology teams of NCAs is required to assess if these findings are generalisable. The heterogeneity of outcomes collected and differences in the degree of missing data will likely lead to this proxy having varying utility across different datasets.

## Conclusion

For the first time, we have demonstrated that poor engagement in a national audit program directly correlates with the quality of care provided. Acknowledging methodological limitations of this work, the key message is unsurprising. Centre-level variation in care is frequently overlooked in observational studies [22], yet any epidemiologist working in the field will recognise that centre effects are large and significant. As research into health care quality evolves, our data suggest that failing to return outcome data is a viable service quality metric.

## Data Availability

Data available via application to HQIP

## Abbreviations

NCAREIA: National Clinical Audit for Rheumatoid and Early Inflammatory Arthritis
RA: Rheumatoid arthritis
NCAs: National clinical audits
SSNAP: Sentinel Stroke National Audit Programme
NHS: National Health Service
HQIP: Healthcare Quality Improvement partnership
EIA: Early inflammatory arthritis
RF: Rheumatoid factor
anti-CCP: Anti-cyclic citrullinated protein
IMD: Index of multiple deprivation
LSOAs: Lower super output areas
ESR: Erythrocyte sedimentation rate
CRP: C-reactive protein
DAS28: Disease activity score
CI: Confidence intervals
SD: Standard deviation
VAS: Visual analogue scale
